# The Survival Benefit and Safety of Splenectomy for Gastric Cancer With Total Gastrectomy: Updated Results_†_

**DOI:** 10.3389/fonc.2020.568872

**Published:** 2021-01-07

**Authors:** Kun Yang, Zhi-Yun Zang, Kai-Fan Niu, Li-Fei Sun, Wei-Han Zhang, Yue-Xin Zhang, Xiao-Long Chen, Zong-Guang Zhou, Jian-Kun Hu

**Affiliations:** ^1^ Department of Gastrointestinal Surgery, West China Hospital, Sichuan University, Chengdu, China; ^2^ Laboratory of Gastric Cancer, State Key Laboratory of Biotherapy/Collaborative Innovation Center of Biotherapy and Cancer Center, West China Hospital, Sichuan University, Chengdu, China; ^3^ West China School of Medicine, Sichuan University, Chengdu, China

**Keywords:** gastric cancer, splenectomy, total gastrectomy, survival benefit, safety

## Abstract

**Background:**

Splenectomy was traditionally performed to dissect the splenic hilar lymph nodes. Considering the important functions of spleen, whether splenectomy would bring beneficial to gastric cancer patients is debatable. This meta-analysis aimed to make an updated evaluation on the effectiveness and safety of splenectomy.

**Methods:**

Literature searches were performed to identify eligible RCTs concerning effectiveness or safety of splenectomy with gastrectomy from PubMed, MEDLINE, CBMdisc, EMBASE, and Cochrane Central Register of Controlled Trials. Two reviewers completed the study selection, data extraction, and quality assessment independently. The meta-analyses were performed by RevMan 5.3.

**Results:**

A total of 971 patients from four studies were included (485 in splenectomy group and 486 in spleen preservation group). Splenectomy did not increase 5-year overall survival rate (RR=1.05, 95% CI: 0.96, 1.16) or increase postoperative mortality (RR=1.21, 95% CI: 0.41, 3.54). However, the analysis demonstrated that gastrectomy with splenectomy had significantly higher incidence of postoperative complications (RR=1.80, 95% CI: 1.33, 2.45). No significant differences were found in terms of the number of resected lymph nodes and reoperation rate; however, splenectomy had a tendency to prolong the duration of surgery and hospital stays. Subgroup analyses indicated that splenectomy could not increase overall survival rate for either whole or proximal gastric cancer. Sensitivity analyses also found similar results compared to the primary analyses.

**Conclusions:**

Splenectomy cannot benefit the survival of patients with tumor located at lesser curvature, and it could instead increase postoperative morbidity.

## Introduction

Gastric cancer is one of the most common malignant tumors, especially in East Asia (1, 2). The gastric cancer treatment guideline suggest gastrectomy with D2 lymph nodes (LNs) dissection to be the standard operation for advanced gastric cancer (3). Traditionally, splenectomy was performed to dissect the splenic hilar LNs (No.10 LNs) since the incidence of lymph nodes metastasis could be up to 26% and spleen-preserving No.10 LNs dissection was technical-demanding (4, 5). As the development of surgical skills and instruments, however, spleen-preserving No.10 LNs dissection has been performed widely. Meanwhile, results about the effectiveness of splenectomy on survival of patients were not confirmed. Most of the retrospective studies failed to show survival advantage of splenectomy compared with spleen preservation, but increased complications (6–8). However, these studies might be strongly biased since the patients who had undergone gastrectomy with splenectomy often had larger tumor, deeper serosa invasion, higher lymph nodes metastatic rate, and more advanced stage (9). On one hand, there were some investigators indicating that gastrectomy plus splenectomy was associated with higher survival rate than gastrectomy alone, although there was no statistically significant difference (10–12). These trials were not yet considered powerful enough to be conclusive (13). On the other hand, splenectomy may result in serious immunological consequences as the spleen is an essential part of the immune system (14). Therefore, whether splenectomy could bring benefit to the patients with gastric cancer is still under debate.

To answer this question, we have performed a meta-analysis to investigate the effectiveness and safety of splenectomy for gastric carcinoma 10 years ago, and we found that splenectomy with total gastrectomy showed a better trend for long-term survival although no significant difference (9). According to the handbook of Cochrane Collaboration, a regular update aiming to find new evidences to integrate into a previous meta-analysis is very important and should in theory be conducted at least twice-yearly, especially an update that might result in an alteration to the results on every occasion (15). As the results of JCOG 0110 randomized trial with largest scale were published and showed an opposite trend, we aimed to make an updated evaluation about the impact of splenectomy on long-term survival, postoperative morbidity, and mortality in gastric cancer patients by adding new publications in the past 10 years.

## Materials and Methods

### Literature Search Strategies

Search strategies were conducted in databases, including PubMed, the Cochrane Central Register of Controlled Trials, Chinese Biomedical Database (CBMdisc), MEDLINE, and EMBASE. Controlled vocabulary and syntax such as Medical subject headings (MeSH) was applied to search corresponding databases, while keywords were also applied. The details of search strategies have been described in our previous meta-analysis (9). The time-frame of this updated literature searching was from December 2008 to April 2020 with no limitations on languages.

### Eligibility Criteria

Inclusion criteria for eligible randomized controlled trials (RCTs) comparing the efficacy or safeness of gastrectomy with splenectomy to that of gastrectomy alone were as follows: (1) patients diagnosed with gastric cancer preoperatively by gastroscopy and biopsy without distant metastasis; (2) patients with resectable primary gastric tumor and were able to tolerate operation; (3) patients received intervention of either curative or palliative gastrectomy; (4) studies reported at least one of the following outcome: prognosis (5-year overall survival rate), safety (postoperative mortality rate and postoperative morbidity rate), number of resected lymph nodes, length of the operation, length of hospital stays and reoperation rate; (5) the splenectomy was performed prophylactically for the dissection of splenic hilar lymph nodes. There were no limitations on patients’ age, gender, race, tumor location, and stage.

Studies were excluded according to the following exclusion criteria: (1) patients with direct invasion of cancer into the pancreas or spleen, gross involvement of the gastrosplenic ligament and macroscopic lymph node metastasis in the splenic hilum or along the splenic artery; (2) patients with other types of gastric tumor (such as lymphoma), tumor on other organs, or gastric tumor with multiple components (such as adenosquamous carcinoma); (3) trials with unequal or uncertain characteristics between groups at baseline; (4) studies did not provide sufficient outcome (studies in which interested outcome was difficult to be calculated from the reported results); (5) patients received combined resection other than splenectomy (such as pancreatosplenectomy); (6) patients treated with chemotherapy, radiotherapy, or immunotherapy perioperatively; (7) review articles, case reports, comments, and editorials/letters.

### Selection and Data Extraction

Prescreening of title, abstract and keywords, and data extraction were conducted by two reviewers independently. Full texts were retrieved and further evaluated during secondary screening if the title and abstract clued that the study could be potentially eligible according to the preset inclusion and exclusion criteria. Afterward, two reviewers completed the final selection of studies.

The data including sample of study (number of patients in each group), interventions and comparators (details of splenectomy and splenic preservation for each group, amount and reason for dropouts and withdrawals if any, along with characteristics of the patients involved), and outcome (e.g., 5-year overall survival rate, postoperative morbidity and mortality rate, and operation-related parameters) were extracted. Furthermore, the country and year of included studies, details of randomization allocation concealment, and blinding assessment, as well as intention to treat analysis, were extracted. If only survival curves were reported in a trial, data from the curve were extracted and converted into overall 5-year survival rate (16). The results of the data that could not be extracted for meta-analysis were presented in a descriptive and qualitative manner.

If a trial reported medians and ranges instead of means and standard deviations, we calculated the means and standard deviations (SD) based on the sample sizes. If neither ranges nor any other dispersion measurements were reported, we used the first and third quartiles to calculate the means and standard deviations (17).

### Quality Assessment

Seven elements were evaluated for quality assessment: (1) random allocation; (2) allocation concealment; (3) baseline equality associated with prognostic characteristics between the two groups; (4) eligible inclusion/exclusion criteria; (5) blinding assessment; (6) details of loss to follow-up in each group; (7) intention-to-treat analysis. Trials including at least 6 of the elements stated above were considered of high quality, those with at least 4 of fair quality, and those including less than 3 of low quality (18).

Any discrepancies in data extraction and quality assessment were debated and solved by a consensus meeting of the reviewers.

### Outcome

Primary outcome included prognosis (5-year overall survival rate) and safety (postoperative mortality rate and postoperative morbidity rate), whereas secondary outcome contained operation-related parameters, including number of resected lymph nodes, length of the operation, duration of hospital stays, and reoperation rate.

### Statistical Analysis

Data analyses were performed by RevMan 5.3. Weighted estimates of relative risks (RR) with 95% confidence intervals (CI) were applied to calculate dichotomous data, while weighted mean differences (WMD) with 95% CI were used to calculate continuous data. A *P-*value of less than 0.050 was considered statistically significant. Heterogeneities of treatment effects between trials were examined using Chi-squared test, for which a *P-*value less than 0.100 was considered statistically significant. Total variation among studies was assessed by I-square (>50%: high heterogeneity, 25% to 50%: moderate heterogeneity, and <25%: low heterogeneity) (19). If heterogeneities presented, one of the following techniques was taken to give a corresponding explanation: (1) random effect model was applied for pooled analysis; (2) subgroup analyses were performed stratified by the longitudinal tumor location, curative degree, and stage; (3) sensitivity analyses were performed only in trials with high quality to avoid bias (20), or by separating the patients with clear circumferential tumor location from other patients without clear circumferential tumor location.

## Results

### Included Literature

**Figure 1:**
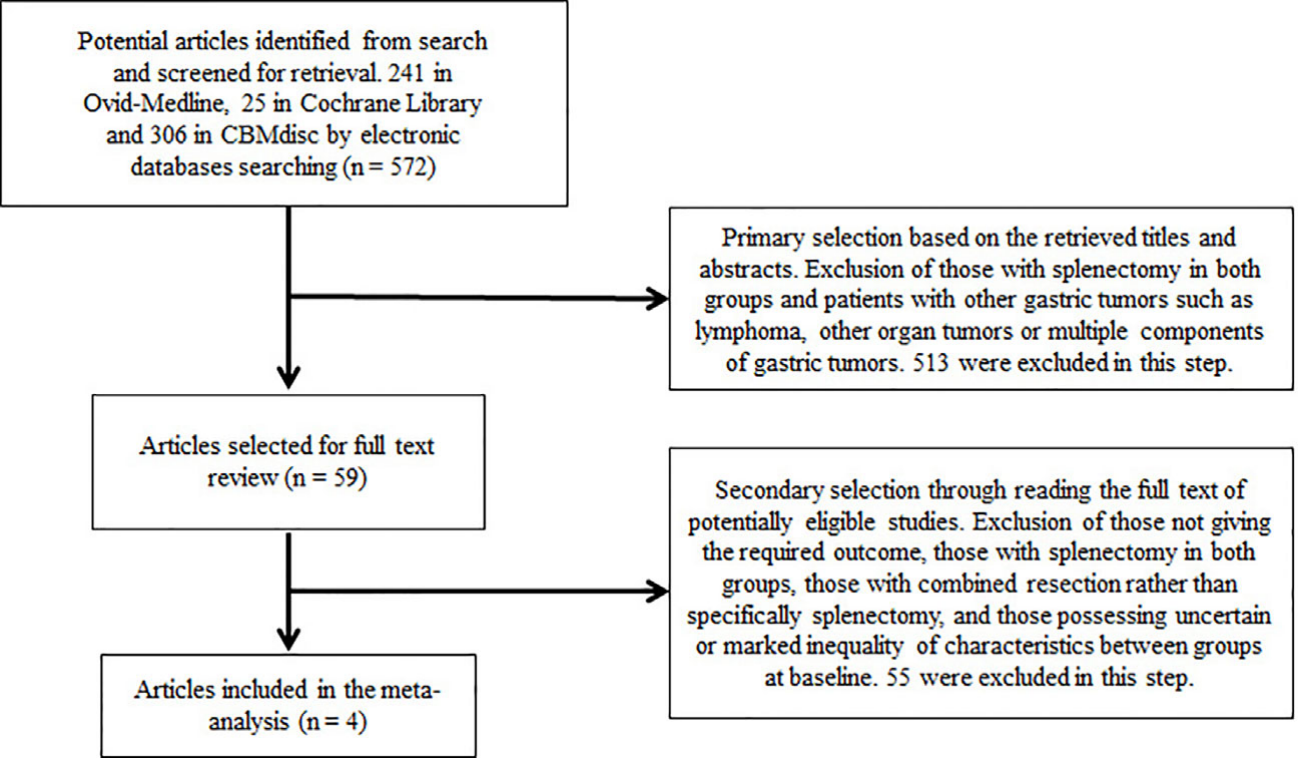
Flow chart showing study selection procedure.

After initial database searching, a total of 572 articles were recognized from the searched electronic databases (241 in Medline, 25 in Cochrane Library, and 306 in CBMdisc without additional findings in other databases). In primary selection, 59 potential eligible articles were retrieved for full-text assessment after screening titles and abstracts, and 513 studies were excluded according to the inclusion/exclusion criteria stated above. In secondary selection, 55 trials were further excluded after reviewing full texts. The selecting procedure and details of excluded studies are summarized in the flowchart (**Figure 1**).

As a result, only four RCTs (10–13) comparing the effectiveness and safety of gastrectomy with splenectomy to that without splenectomy in patients with histologically confirmed gastric cancer were included. A total of 971 patients were enrolled in the analyses, with 485 patients receiving gastrectomy plus splenectomy while 486 patients were assigned to gastrectomy alone. The characteristics and study quality of the included RCTs are showed below (**Tables 1**, **2**).

**Table 1:** The characteristics of the included RCTs.

Study	Participants	Interventions	Outcome
Toge et al. (10)	“The patients underwent total gastrectomy with tumor located on lesser curvature. Patients were allocated into 2 groups at random: 41 in splenectomy group and 38 in spleen preservation group.” (9)	“Splenectomy *vs* splenic preservation. The follow-up was at least 5 years” (9) Splenic hilar nodes were removed in spleen preservation group.	“Kaplan-Meier survival curve. 5-year overall survival were from reported percentages data.” (9)
Yu et al. (11)	“A total of 216 patients with proximal gastric cancer were randomized. 103 patients had the spleen preserved and 104 had a splenectomy.” (9)	“Splenectomy *vs* splenic preservation. Of the 207 patients, 7 were lost to follow up (follow-up rate 96.6%) and mean duration of follow-up was 5.4 years.” (9) Splenic hilar nodes were removed in spleen preservation group.	“Harvested lymph nodes. Postoperative morbidity and mortality. Kaplan-Meier survival curve. 5-year overall survival were from reported percentages data.” (9)
Csendes et al. (12)., 2002	“187 patients with gastric carcinoma entered this study. 97 patients with total gastrectomy and 90 patients with total gastrectomy and splenectomy.” (9)	“Total gastrectomy *vs* total gastrectomy plus splenectomy. The follow-up was at least 5 years.” (9) Splenic hilar nodes were removed in spleen preservation group.	“Five-year overall survival and survival by stage. Postoperative morbidity and mortality. Kaplan-Meier survival curve. Duration of operation and hospital stay.” (9)
Sano et al. (13)	Proximal gastric adenocarcinoma of T2-4/N0-2/M0 without invading the greater curvature and macroscopic No.10 and 11d lymph node metastasis was eligible. 505 patients were randomly assigned to receive either total gastrectomy with splenectomy (254 patients) or without splenectomy (251patients).	Total gastrectomy with splenectomy *vs* Total gastrectomy without splenectomy. The follow-up was performed in a median period of 71.8 months. The nodes along the splenic artery were dissected; however, splenic hilar nodes were removed optionally without splenic mobilization.	Five-year overall survival. Five-year relapse-free survival. Postoperative morbidity and mortality. Kaplan-Meier survival curve. Duration of operation, blood loss and retrieved nodes.

**Table 2:** The quality of the included randomized trials.

Study	Truly random	Concealed allocation	Baseline features	Eligibility criteria	Blinding assessment	Loss to follow-up	Intention to treat	Study quality
Toge et al. (10)	Unclear	Unclear	No	No	Unclear	Unclear	Unclear	Poor
Yu et al. (11)	Yes	Unclear	Yes	Yes	Unclear	Yes	Unclear	Fair
Csendes et al. (12)	Yes	Unclear	Yes	Yes	Unclear	Yes	No	Fair
Sano et al. (13)	Yes	Yes	Yes	Yes	Unclear	Yes	Yes	High

### Effectiveness

**Figure 2:**
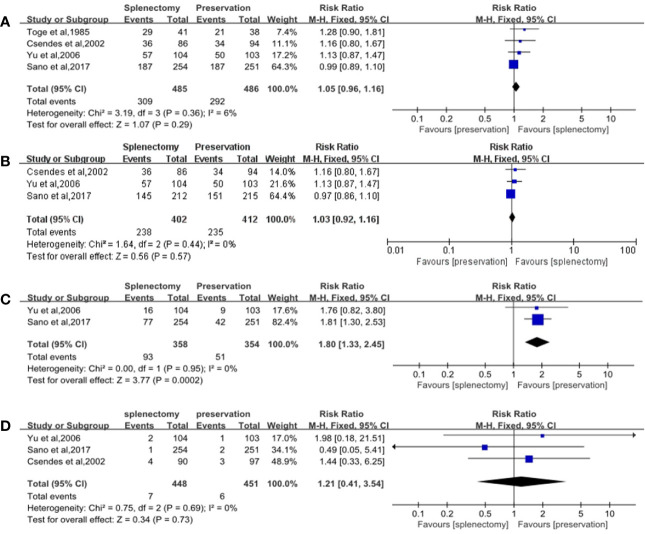
Forest plots of overall survival rates and safeties. **(A)** Forest plot of 5-year overall survival rate for all cases, with 95%CI. Data for a fixed-effects model are shown as there was no statistical heterogeneity; **(B)** Forest plot of 5-year overall survival rate for cases with proximal and whole gastric carcinoma, with 95%CI. Data for a fixed-effects model are shown as there was no statistical heterogeneity; **(C)** Forest plot of postoperative morbidity, with 95%CI. Data for a fixed-effects model are shown as there was no statistical heterogeneity; **(D)** Forest plot of postoperative mortality, with 95%CI. Data for a fixed-effects model are shown as there was no statistical heterogeneity.

The number of alive patients was used as the number of events to analyze the effectiveness between splenectomy group and splenic preservation group. No significant difference was showed between gastrectomy with splenectomy and gastrectomy without splenectomy on 5-year overall survival rate, with RR of 1.05 (95% CI: 0.96, 1.16) (**Table 3**, **Figure 2A**).

**Table 3:** Outcome of overall survival rates, safety, operation-related events and subgroup analysis stratified by the longitudinal tumor location.

	No. of studies	Splenectomy(n^*^/N)	Splenic preservation(n^*^/N)	RR/WMD(95% CI)	*P*-value for effect size	*P*-value for heterogeneity	Effect model
Overall survival rate	4	309/485	292/486	1.05(0.96,1.16)	0.290	0.360	Fixed
Postoperative morbidity	2	93/358	51/354	1.80(1.33,2.45)	0.0002	0.950	Fixed
Postoperative mortality	3	7/448	6/451	1.21(0.41,3.54)	0.730	0.690	Fixed
Operation time(min)	2	344^†^	348^†^	7.86(-0.83,16.54)	0.080	0.760	Fixed
Duration of hospital stay(d)	1	90^†^	97^†^	3.20 (-0.00, 6.40)	0.050	NA	Fixed
No. of harvested lymph nodes	2	358^†^	354^†^	2.50(-2.40,7.40)	0.320	0.090	Random
Reoperation	2	13/344	13/348	1.05(0.50,2.20)	0.890	0.580	Fixed
Overall survival rate stratified by location of tumor (whole and proximal stomach)	3	238/402	235/412	1.03 (0.92, 1.16)	0.570	0.440	Fixed
Overall survival rate for patients with curative gastrectomy	4	294/431	283/432	1.04 (0.95, 1.14)	0.440	0.350	Fixed

*Represents the patients alive; ^†^The summed number of patients in each group; RR, Relative risk; WMD, Weighted mean differences; CI, Confidence interval; NA, Not applicable.

Subgroup analyses were performed stratified by the longitudinal tumor location and stage, due to the fact that they were important factors determining whether splenectomy for hilar lymphadenectomy was needed. In the subgroup analyses of longitudinal tumor location, we found that splenectomy could not improve patients’ survival for those with whole and proximal gastric cancer. The RR of 5-year overall survival rate was 1.03 (95% CI: 0.92, 1.16), which did not suggest any significant differences between splenectomy and spleen preservation for patients with whole and proximal gastric cancer (**Table 3**, **Figure 2B**). The results of subgroup analyses stratified by the curative degree showed that the overall survival rates between the splenectomy group and spleen preservation group were not significant different with the RR of 5-year overall survival rate being 1.04 (95% CI: 0.95, 1.14) for patients with curative gastrectomy.

For overall survival rate stratified by stage, only one RCT was included for this analysis (12). There were no significant differences between the two groups on 5-year overall survival rates in patients with stage I, stage II, and stage III (all *P-*values > 0.050). Another RCT stratified by T and N stage (13) revealed that no significant differences were found on survival rates between total gastrectomy with splenectomy and total gastrectomy alone.

### Safety

Postoperative morbidity was significantly higher in splenectomy group than spleen preservation group with RR of 1.80 (95% CI: 1.33, 2.45) (**Table 3**, **Figure 2C**). However, no significant difference was found in terms of mortality between the two groups, with RR of 1.21 (95% CI: 0.41, 3.45) (**Table 3**, **Figure 2D**).

### Operation-Related Parameters

For number of retrieved lymph nodes, no significant difference was demonstrated between the two groups with WMD of 2.50. Furthermore, splenectomy trended to prolong the length of operation and duration of hospital stays (WMD = 7.86 min, *P*=0.080 and 3.20 d, *P*=0.050). Regarding the reoperation rate, there was no significant difference between the two groups (RR=1.05, 95% CI: 0.50, 2.20) (**Table 3**).

### Sensitivity Analysis

After excluding trials with low quality, the results of sensitivity analysis showed that no significant differences were observed between the two groups in terms of 5-year overall survival rates and postoperative mortality (RR = 1.03 and 1.21, respectively). However, there was a statistical difference in terms of postoperative morbidity between the two groups (RR=1.80, 95% CI: 1.33, 2.45) (**Table 4**). After separating the patients with clear circumferential tumor location from other patients without clear circumferential tumor location, splenectomy could still not improve patients’ survival, neither in those with gastric cancer located at the lesser curvature (RR=1.02, 95% CI: 0.92, 1.13) nor in patients without clear circumferential tumor location (RR=1.14, 95% CI: 0.92, 1.41).

**Table 4:** Sensitivity results of overall survival rates and safety.

	No. of studies	Splenectomy(n^*^/N)	Splenic preservation(n ^*^/N)	RR/WMD(95% CI)	*P*-value for effect size	*P*-value for heterogeneity	Effect model
Excluding trials with low quality							
Overall survival rate	3	280/444	271/448	1.03(0.94,1.14)	0.500	0.460	Fixed
Postoperative morbidity	2	93/358	51/354	1.80(1.33,2.45)	0.0002	0.950	Fixed
Postoperative mortality	3	7/448	6/451	1.21(0.41,3.54)	0.730	0.690	Fixed
Patients with clear circumferential tumor location (cancer located at the lesser curvature)							
Overall survival rate	2	216/295	208/289	1.02(0.92,1.13)	0.72	0.16	Fixed
Patients without clear circumferential tumor location							
Overall survival rate	2	93/190	84/197	1.14(0.92,1.41)	0.23	0.91	Fixed

## Discussion

Gastric cancer is still rampant in East Asian countries, although its global incidence has declined (1, 2). On the other hand, incidence of adenocarcinoma in the upper third of the stomach increased over the last 2 decades worldwide (21–23). Since the frequency of LNs metastasis depends on tumor location and stage, the splenic hilar LNs have a higher metastatic rate in proximal gastric and gastroesophageal junction cancer. Therefore, splenic hilar LNs were required to dissect when performing total gastrectomy (3). Recently, the results of the JCOG 0110 trial showed that the overall survival of spleen-preserving group with selective splenic hilar LNs dissection was not lower than that of splenectomy group with fully removal of splenic hilar LNs. Consequently, the dissection of No.10 LNs was waived from the extent of D2 lymphadenectomy in total gastrectomy in the latest version of Japanese guideline (13, 24). We should note, however, only tumors located in the lesser curvature and relative early stage were enrolled in this trial. Moreover, the likelihood of residual disease in splenic hilar LNs might increase if No.10 lymphadenectomy was not done, since preoperative radiological modalities were not accurate enough to foresee splenic LNs metastasis. Furthermore, No.10 lymphadenectomy might be helpful and essential for some patients with positive splenic hilar LNs or having high metastatic risk (25). In fact, it has been reported that a 10% difference on 5-year survival rate was detected between the groups with and without splenic hilar lymphadenectomy, although not statistically different (26). Therefore, splenic hilar LNs dissection in total gastrectomy should not be abolished completely, and its prognostic value is still debatable. Nevertheless, the necessity of splenectomy for the purpose of hilar LNs dissection remains controversial.

In this meta-analysis, we did not find significant difference in terms of 5-year overall survival rate between the two groups. After stratified by the longitudinal location, a similar result was observed. In the sensitivity analysis, gastrectomy plus splenectomy showed no clear survival advantage over gastrectomy alone for all cases and cases with whole and proximal gastric cancer. Even for those with positive No.10 LNs or LNs along the splenic artery, splenectomy could not improve survival (11, 27). Compared to the previous meta-analysis, our updated analysis showed similar results. However, the newly included JCOG0110 trial found that spleen preservation could show a positive trend for survival (13), while the other included studies indicated an opposed trend (10–12). This inconsistency in the overall survival may derive from ethnical heterogeneity, varied stage of included patients, different extent of lymphadenectomy and application of adjuvant chemotherapy, etc. as these studies were conducted in different periods.

Although the splenic hilar nodes were removed optionally in the spleen preservation group of newly included study (JCOG 0110 trial), only 1.58% (8/505) patients had histological metastasis in No.10 nodes. The results of our previous published meta-analysis, which included the other three trials with dissection of No.11d and No.10 LNs in spleen preservation group, have found that splenectomy had no significant influence on survival rates (9). Meanwhile, the latest version of Japanese gastric cancer treatment guideline has cancelled the No.10 nodes in the D2 dissection of total gastrectomy (28). Therefore, we considered that the oncological outcome would not be affected in this meta-analysis.

We included patients with palliative gastrectomy because: (1) We can find the related studies as complete as possible. For a meta-analysis, including all related studies is very important. (2) Since this manuscript was an update to our previous meta-analysis, we needed to keep the inclusion criteria accordant. (3) Although the four included trials were RCTs and well conducted, they inevitably had patients with palliative gastrectomy in each group, which has been clearly reported in the studies. If we excluded the studies with palliative gastrectomy, there would be no eligible study. Notably, patients with palliative gastrectomy in each study were few and balanced in distribution in splenectomy group and spleen preservation group. Therefore, the probability of overall survival rates being affected was low. Furthermore, we have performed the subgroup analysis to investigate the effectiveness of splenectomy only in the patients with curative gastrectomy (**Table 3**), and the results of subgroup analysis were similar to the primary analysis.

Although two studies did not have data concerning the circumferential location of gastric cancer (11, 12), another two studies included patients with gastric cancer located at the lesser curvature, which had more than half of the enrolled patients, and accounted for the large part of analysis results (10, 13). After separating the patients with clear circumferential tumor location from other patients without clear circumferential tumor location, splenectomy could not improve patients’ survival for those with gastric cancer located at the lesser curvature. And for patients without clear circumferential tumor location, no matter located at the lesser curvature or greater curvature, there were still no significant differences in terms of overall survival between splenectomy group and splenic preservation group. Based on this, we can at least conclude that splenectomy could not bring benefit to the patients with tumor located at lesser curvature. Regarding the gastric cancer invading the greater curvature of the upper stomach, the fourth edition of Japanese treatment guideline recommended that complete clearance of No.10 nodes by splenectomy should be considered for potentially curable T2-T4 tumors (3). However, the fifth edition indicated that whether splenectomy had an oncological benefit for such cases remains equivocal (28). This meta-analysis still had no answer to this question. Further studies evaluating the effectiveness of splenectomy for gastric cancer located at the upper third with invading the greater curvature are still needed.

Although some early trials showed that splenectomy can lead to higher postoperative mortality (29–31), the present meta-analysis failed to show any significant differences on postoperative mortality, which was confirmed in the sensitivity analysis. However, our meta-analysis showed that gastrectomy with splenectomy was likely to be associated with increased postoperative morbidity, which was in accordance with the previous research (6, 8, 29–31). In our previous meta-analysis, we found that splenectomy might be related to the increased postoperative morbidities, such as pancreatic leakage, pancreatitis, abdominal abscess, anastomotic leakage, ileus and pleural effusion (32). In addition, the results of our previous meta-analysis also showed that splenectomy could not harvest more lymph nodes but tended to increase the duration of surgery and duration of hospital stays instead.

To guarantee the reliability and validity of the results, we included only RCTs to strengthen the evidence for prognosis between gastrectomy with and without splenectomy in gastric cancer patients. Therefore, there were only limited research studies to analyze. Additionally, the sample sizes were relatively small for the pooled analyses, which could add uncertainty of generalizability to the results. Indeed, we have carefully selected some non-RCTs with good balanced baseline characteristics for meta-analysis once (9), and we found the same results as those of RCTs. Therefore, the conclusion could be drawn based on the results of RCTs and non-RCTs. Third, the recurrence was not calculated in this meta-analysis because only one study reported the recurrence data, and overall survival was more important and valuable than disease-free survival on reflecting patients’ prognosis. Therefore, overall survival should be the primary outcome. Although the study has limitations as stated above, great efforts had been made to minimize the probability of biases through designing a detailed research plan, conducting a comprehensive search, applying objective approaches for selecting studies, extracting and analyzing data, and performing subgroup and sensitivity analyses (9).

## Conclusion

Splenectomy cannot benefit the survival of patients with tumor located at lesser curvature, and it could instead increase postoperative morbidity.

## Data Availability Statement

The original contributions presented in the study are included in the article/supplementary materials. Further inquiries can be directed to the corresponding authors.

## Author Contributions

Conception or design of the work: KY, Z-GZ, J-KH. Acquisition, analysis, or interpretation of data for the work: KY, Z-YZ, K-FN, L-FS, W-HZ, Y-XZ, X-LC, Z-GZ, J-KH. Drafting the work or revising it critically for important intellectual content: KY, Z-YZ, K-FN. Final approval of the version to be published: KY, J-KH. All authors contributed to the article and approved the submitted version.

## Funding

This work was supported by the National Natural Science Foundation of China [No. 81772547]; the Fundamental Research Funds for the central Universities [No. 2017SCU04A18]; Young scientific and academic leaders training program of Sichuan University [No. 0082604151001/035]; Foundation of Science & Technology Department of Sichuan Province [No. 2019YFS0256]; and 1. 3. 5 project for disciplines of excellence, West China Hospital, Sichuan University [No. ZY2017304].

## Conflict of Interest

The authors declare that the research was conducted in the absence of any commercial or financial relationships that could be construed as a potential conflict of interest.

## References

[1] Siegel RL Miller KD Jemal A Cancer statistics, 2018. CA Cancer J Clin (2018) 68(1):7–30. 10.3322/caac.21442 29313949

[2] Bray F Ferlay J Soerjomataram I Siegel RL Torre LA Jemal A Global cancer statistics 2018: GLOBOCAN estimates of incidence and mortality worldwide for 36 cancers in 185 countries. CA Cancer J Clin (2018) 68(6):394–424. 10.3322/caac.21492 30207593

[3] Japanese Gastric Cancer Association Japanese gastric cancer treatment guidelines 2014 (ver. 4). Gastric Cancer (2017) 20(1):1–19. 10.1007/s10120-016-0622-4 PMC5215069 27342689

[4] Ishikawa S Shimada S Miyanari N Hirota M Takamori H Baba H Pattern of lymph node involvement in proximal gastric cancer. World J Surg (2009) 33(8):1687–92. 10.1007/s00268-009-0083-6 19488813

[5] Hyung WJ Lim JS Song J Choi SH Noh SH Laparoscopic spleen-preserving splenic hilar lymph node dissection during total gastrectomy for gastric cancer. J Am Coll Surg (2008) 207:e6–11. 10.1016/j.jamcollsurg.2008.04.027 18656040

[6] Ohkura Y Haruta S Shindoh J Tanaka T Ueno M Udagawa H Efficacy of prophylactic splenectomy for proximal advanced gastric cancer invading greater curvature. World J Surg Oncol (2017) 15(1):106. 10.1186/s12957-017-1173-9 28545537 PMC5445509

[7] Son SY Shin DJ Park YS Oo AM Jung DH Lee CM Spleen-preserving lymphadenectomy versus splenectomy in laparoscopic total gastrectomy for advanced gastric cancer. Surg Oncol (2017) 26(2):207–11. 10.1016/j.suronc.2017.04.002 28577727

[8] Wang Q Dang T Meng X Li K Ren W Ma X Is concomitant splenectomy necessary in radical gastric cancer surgery? A systematic review and meta-analysis. Asia Pac J Clin Oncol (2018) 15(2):e28–35. 10.1111/ajco.13052 30178572

[9] Yang K Chen XZ Hu JK Zhang B Chen ZX Chen JP Effectiveness and safety of splenectomy for gastric carcinoma: a meta-analysis. World J Gastroenterol (2009) 15(42):5352–9. 10.3748/wjg.15.5352 PMC2776865 19908346

[10] Toge T Kameda A Kuroi K Seto Y Yamada H Hattori T The role of the spleen in immunosuppression and the effects of splenectomy on prognosis in gastric cancer patients. Nippon Geka Gakkai Zasshi (1985) 86:1120–3. 4088224

[11] Yu W Choi GS Chung HY Randomized clinical trial of splenectomy versus splenic preservation in patients with proximal gastric cancer. Br J Surg (2006) 93:559–63. 10.1002/bjs.5353 16607678

[12] Csendes A Burdiles P Rojas J Braghetto I Diaz JC Maluenda F A prospective randomized study comparing D2 total gastrectomy versus D2 total gastrectomy plus splenectomy in 187 patients with gastric carcinoma. Surgery (2002) 131:401–7. 10.1067/msy.2002.121891 11935130

[13] Sano T Sasako M Mizusawa J Yamamoto S Katai H Yoshikawa T Randomized Controlled Trial to Evaluate Splenectomy in Total Gastrectomy for Proximal Gastric Carcinoma. Ann Surg (2017) 265(2):277–83. 10.1097/SLA.0000000000001814 27280511

[14] Llende M Santiago-Delpín EA Lavergne J Immunobiological consequences of splenectomy: a review. J Surg Res (1986) 40(1):85–94. 10.1016/0022-4804(86)90149-6 3510337

[15] Higgins JPT Thomas J Chandler J Cumpston M Li T Page MJ. eds. Cochrane Handbook for Systematic Reviews of Interventions. 2nd Edition. Chichester (UK): John Wiley & Sons, (2019).

[16] Parmar MK Torri V Stewart L Extracting summary statistics to perform meta-analyses of the published literature for survival endpoints. Stat Med (1998) 17(24):2815–34. 10.1002/(SICI)1097-0258(19981230)17:24<2815::AID-SIM110>3.0.CO;2-8 9921604

[17] Wan X Wang W Liu J Tong T Estimating the sample mean and standard deviation from the sample size, median, range and/or interquartile range. BMC Med Res Methodol (2014) 14:135. 10.1186/1471-2288-14-135 25524443 PMC4383202

[18] NHS Centre for Reviews and Dissemination Undertaking systematic reviews of research on effectiveness: CRD"s guidance for carrying out or commissioning reviews (Report No 4). 2nd ed York: NHS CRD (2001).

[19] Higgins JP Thompson SG Deeks JJ Altman DG Measuring inconsistency in meta-analyses. BMJ (2003) 327:557–60. 10.1136/bmj.327.7414.557 PMC192859 12958120

[20] Dixon E Hameed M Sutherland F Cook DJ Doig C Evaluating meta-analyses in the general surgical literature: a critical appraisal. Ann Surg (2005) 241:450–9. 10.1097/01.sla.0000154258.30305.df PMC1356983 15729067

[21] Colquhoun A Arnold M Ferlay J Goodman KJ Forman D Soerjomataram I Global patterns of cardia and non-cardia gastric cancer incidence in 2012. Gut (2015) 64(12):1881–8. 10.1136/gutjnl-2014-308915 25748648

[22] Liu K Yang K Zhang W Chen X Chen X Zhang B Changes of Esophagogastric Junctional Adenocarcinoma and Gastroesophageal Reflux Disease Among Surgical Patients During 1988-2012: A Single-institution, High-volume Experience in China. Ann Surg (2016) 263(1):88–95. 10.1097/SLA.0000000000001148 25647058 PMC4679348

[23] Ahn HS Lee HJ Yoo MW Jeong SH Park DJ Kim HH Changes in clinicopathological features and survival after gastrectomy for gastric cancer over a 20-year period. Br J Surg (2011) 98(2):255–60. 10.1002/bjs.7310 21082693

[24] Japanese Gastric Cancer Association Gastric cancer treatment guidelines 2018. Tokyo: Kanehara Press (2018), ISBN: 978-4-307-20381-4.

[25] Chen XL Yang K Zhang WH Chen XZ Zhang B Chen ZX Metastasis, risk factors and prognostic significance of splenic hilar lymph nodes in gastric adenocarcinoma. PLoS One (2014) 9:e99650. 10.1371/journal.pone.0099650 24915065 PMC4051839

[26] Yang K Zhang WH Chen XZ Chen XL Zhang B Chen ZX Survival benefit and safety of no. 10 lymphadenectomy for gastric cancer patients with total gastrectomy. Med (Baltimore) (2014) 93(25):e158. 10.1097/MD.0000000000000158 PMC4616371 25437029

[27] Zhang CH Zhan WH He YL Chen CQ Huang MJ Cai SR Spleen preservation in radical surgery for gastric cardia cancer. Ann Surg Oncol (2007) 14:1312–9. 10.1245/s10434-006-9190-x 17265118

[28] Japanese Gastric Cancer Association Japanese gastric cancer treatment guidelines 2018 (5th edition). Gastric Cancer (2020). 10.1007/s10120-020-01042-y PMC7790804 32060757

[29] Bonenkamp JJ Hermans J Sasako M van de Velde CJ Welvaart K Songun I Extended lymph-node dissection for gastric cancer. N Engl J Med (1999) 340:908–14. 10.1056/NEJM199903253401202 10089184

[30] Cuschieri A Fayers P Fielding J Craven J Bancewicz J Joypaul V Postoperative morbidity and mortality after D1 and D2 resections for gastric cancer: preliminary results of the MRC randomised controlled surgical trial. The Surgical Cooperative Group. Lancet (1996) 347:995–9. 10.1016/S0140-6736(96)90144-0 8606613

[31] Bonenkamp JJ Songun I Hermans J Sasako M Welvaart K Plukker JT Randomised comparison of morbidity after D1 and D2 dissection for gastric cancer in 996 Dutch patients. Lancet (1995) 345:745–8. 10.1016/S0140-6736(95)90637-1 7891484

[32] Adachi Y Kamakura T Mori M Maehara Y Sugimachi K Role of lymph node dissection and splenectomy in nodepositive gastric carcinoma. Surgery (1994) 116:837–41. 7940186

